# BEaTS-α an open access 3D printed device for *in vitro* electromechanical stimulation of human induced pluripotent stem cells

**DOI:** 10.1038/s41598-020-67169-1

**Published:** 2020-07-09

**Authors:** David Cortes, Christopher D. McTiernan, Marc Ruel, Walfre Franco, Cencen Chu, Wenbin Liang, Erik J. Suuronen, Emilio I. Alarcon

**Affiliations:** 1 Biomedical Mechanical Engineering, University of Ottawa, Ottawa, ON K1N6N5 Canada; 2 Division of Cardiac Surgery, University of Ottawa Heart Institute, Ottawa, ON K1Y4W7 Canada; 3 Wellman Center for Photomedicine, Department of Dermatology, Massachusetts General Hospital, Harvard Medical School, Boston, MA 02114 USA; 4 Cardiac Electrophysiology Laboratory, University of Ottawa Heart Institute, Ottawa, ON K1Y4W7 Canada; 5 Department of Cellular and Molecular Medicine, University of Ottawa, Ottawa, ON K1H8M5 Canada; 6 Biochemistry, Microbiology and Immunology, University of Ottawa, Ottawa, ON K1H8M5 Canada

**Keywords:** High-throughput screening, Biomedical engineering

## Abstract

3D printing was used to develop an open access device capable of simultaneous electrical and mechanical stimulation of human induced pluripotent stem cells in 6-well plates. The device was designed using Computer-Aided Design (CAD) and 3D printed with autoclavable, FDA-approved materials. The compact design of the device and materials selection allows for its use inside cell incubators working at high humidity without the risk of overheating or corrosion. Mechanical stimulation of cells was carried out through the cyclic deflection of flexible, translucent silicone membranes by means of a vacuum-controlled, open-access device. A rhythmic stimulation cycle was programmed to create a more physiologically relevant *in vitro* model. This mechanical stimulation was coupled and synchronized with *in situ* electrical stimuli. We assessed the capabilities of our device to support cardiac myocytes derived from human induced pluripotent stem cells, confirming that cells cultured under electromechanical stimulation presented a defined/mature cardiomyocyte phenotype. This 3D printed device provides a unique high-throughput *in vitro* system that combines both mechanical and electrical stimulation, and as such, we foresee it finding applications in the study of any electrically responsive tissue such as muscles and nerves.

## Introduction

Recent advances in tissue engineering, along with the discovery of induced pluripotent stem cells (iPSC) has opened the doors to new therapies for treating cardiovascular diseases (CVD)^1,2^. In the last decades, researchers have developed and improved differentiation techniques in order to obtain cardiomyocytes derived from human induced pluripotent stem cells (hiPSC-CM)^3,4^. These cells have been used both as therapeutics for the regeneration of lost cardiac tissue as well as in *in vitro* models of cardiac tissues for drug screening^1,5–7^. Although these techniques have great potential as therapies for cardiac tissue regeneration, the standard protocols for differentiation of hiPSC into cardiomyocytes do not provide a combination of electrical and mechanical stimulation, which can limit the maturation of cells^1,5^.

For iPSC to differentiate into cardiomyocytes and reach maturation, soluble factors, cell-to-cell interactions, continuous perfusion, mechanical loading, and electrical stimuli must be present^7–12^. Most recently, iPSC differentiation has been accomplished through the use of soluble factors and chemicals that are added into 2D cell culture. Some researchers have also developed 3-dimensional (3D) cell culture models to further mimic the extracellular matrix (ECM) of cells within the body and have shown that the 3D geometry of such constructs improves the survival and maturation of cells^13,14^. However, despite these advancements, cardiomyocyte maturation remains incomplete, which deems these models less physiologically relevant for drug testing and tissue regeneration techniques^1,6^. Two factors that have been overlooked in the past, and which have only started being incorporated in the last decades, are the mechanical and electrical stimulation that the native cells experience during the beating of the heart^15,16^.

Although there has been a push in the last few decades to develop *in vitro* cell culture models that better mimic the beating of the human heart, most devices stimulate cells either mechanically or electrically^7^. The list of the different approaches taken to provide mechanical stimulation is extensive, but some representative examples include the use of flexible substrates that are stretched using posts^9,11,12,17–20^, magnets^21^, camshafts^18,22^, clamps^23^, and/or vacuum-driven devices^11,17,24,25^. Some of these have been made commercially available such as those developed by Flexcell Inc. (Flexcell International, Hillsborough, NC). Their vacuum-driven devices use flexible, translucent membranes and posts to create cyclic stimulation profiles on the cultured cells. These devices have been used and modified to further develop several stimulation devices for cells under tensile and/or compressive uniaxial and biaxial strain^11,18,22,24–27^. Nevertheless, most of them fail to incorporate electrical pacing and require expensive components that increase the cost of manufacturing and, in some cases, can compromise their reproducibility and their use inside cell incubators.

To address this, some groups have developed stimulation devices that combine both stimulation techniques and have been able to improve the maturation strategies of hiPSC *in vitro*. Kroll *et al*.^1^, developed a device for the electromechanical conditioning of iPSC-derived cardiomyocytes (iPSC-CMs). The cells were cultured in PDMS substrates and assembled onto a custom-made motor-driven apparatus that created cyclic mechanical stress in combination with electrical pacing by means of the commercially available C-PACE system (IonOptix, USA). Ruan *et al*.^6^, developed a technique to condition iPSC-cell derived cardiac tissue. They cultured and differentiated iPSC-CMs to generate a collagen-based bioengineered human cardiac tissue. This artificial tissue was then conditioned under separate mechanical and electrical regimes; after cell culture, differentiation, and conditioning, they observed that the engineered heart tissue was highly responsive to the stimulation techniques. They found that after two weeks of static stress followed by one week of electrical pacing, cell alignment, cardiac hypertrophy, passive stiffness, and contractility was improved. Although effectively incorporating both stimulation regimes, these devices, as well as those developed by other research groups^2,5,28,29^, have shown great variability in the results of the maturation of hiPSC-CMs, determined by protein and gene expression, cell alignment, and electrical conductivity. As many of these observed differences can be attributed to the approaches each of these devices use to accomplish the stimulation, it is evident that the absence of a standard technique, device, and protocol that can be used across research groups hinders straightforward comparison of results.

As such, we set out to develop a readily accessible and cost-effective device able to electro-mechanically stimulate cells, which we foresee being instrumental in the harmonization of cell differentiation protocols amongst laboratories as well as the development of models for the study of stem cell therapies and use in drug screening.

**Figure 1:**
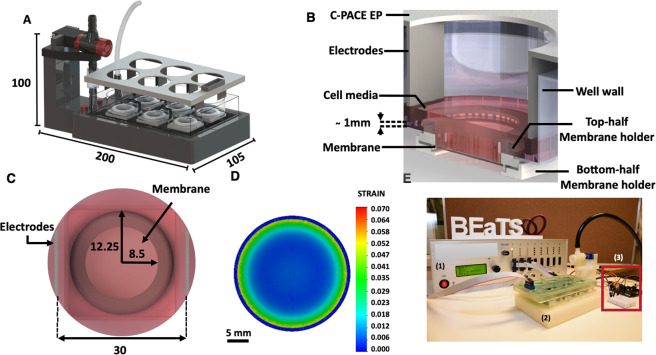
BEaTS-α a 3D Printed device for mechanical and electrical stimulation of cells *in vitro*. (**A**) Parts and components of the 3D printed stimulation device; Standard Tessellation Language (.stl) files for these, and all other 3D printed parts are accessible https://figshare.com/s/504b0f7ccbdc7b893e3a. (**B**) The bottom of the wells of a 6-well plate were removed and replaced by flexible, translucent silicone sheets. Cells were cultured on the membranes and mechanically stimulated by means of a vacuum-driven system. In addition, to simultaneously stimulate cells electrically, a C-PACE EP system was placed over the 6-well plate. The electrodes of the C-PACE EP system were inserted in the media and a TTL-controlled signal was sent to create a physiologically relevant cyclic stimulation cycle. (**C**) Top view of a well with the modified well bottom and the electrodes of the C-PACE EP system. Once assembled, the cells were seeded in a growth area of 2.26 cm^2^. (**D**) Finite Element Analysis (FEA) of the strain generated on the flexible membranes. A pressure of 55 kPa resulted in the deflection of the membranes with an average strain of 2.34%. FEA of the stress (Von Mises) and deflection (URES) profiles are shown in Fig. S1. (**E**) Actual image of the system assembled depicting the electrical impulse generator (1), mechanical stimulator (2), and Arduino UNO interface, red-boxed (3). All dimensions shown are in [mm].

Additive manufacturing, also known as three-dimensional printing (3D printing), is a powerful tool for producing cost-effective devices. In 3D printing, the design of the virtual model using Computer-Aided Design (CAD) software precedes prototype manufacturing. This, followed by optimization of the model and iteration of prototypes, produces novel functional devices with complex geometries at a lower cost. Using this technology, we designed and developed a 3D printed electromechanical stimulation device (BEaTS-α, Fig. 1). The device was designed using CAD software and 3D printed with autoclavable, FDA approved materials. We tested BEaTS-α using human iPSCs to evaluate the effect of each stimulation regime.

## Results and Discussion

The device was designed using CAD software (SolidWorks, Dassault Systems) and 3D printed in an Ultimaker S5 with Nylon 680 filament (Fig. 1A). The design allows the user to culture the cells in standard well-plates, and to make use of the C-PACE EP system for the electrical stimulation of cells, as described below. To achieve the mechanical stimulation, the bottom of each well was removed and replaced with a 3D printed membrane holder as shown in Fig. 1B. Replacing the bottom of each well was a flexible, translucent silicone membrane (Grace Bio-labs, USA) where cells were seeded in a growth area of 2.26 cm^2^ (Fig. 1C); for a representative example of the deflection go here Video S1. To characterize the mechanical stimulation regime on the membranes, a Finite Element Analysis (FEA) was conducted in SolidWorks. The strain profile on the membrane was calculated as shown in Fig. 1D. The average strain on each membrane was 2.34%. The stress (Von Mises) and deflection (URES) profiles were also calculated and shown in Fig. S1. An image of the assembled device is shown in Fig. 1E.

### Stimulation regimes

#### Mechanical stimulation

Mechanical stimulation was accomplished by means of a vacuum-driven system (Fig. 1), an Arduino UNO, an air-control valve, and the in-house designed plate and membrane holders. The deflection of the flexible, translucent membranes, where cells were seeded was accomplished by creating a pressure differential between the inside and the outside of the device. Once the plate was located on the plate holder, the air pump removed the air from within the device creating an inner pressure of 55 kPa. This created a pull on the membrane, generating a strain profile on the membranes which is translated to the cells seeded on top. To calculate the strain generated on the flexible membranes of the stimulation device, a numerical Finite Element Analysis (FEA) was run in SolidWorks. From the FEA on the model of the membrane, it was calculated that at the peak deflection, the maximum, minimum, and average strain were 8.9%, 0.02%, and 2.34%, respectively (Fig. 1D). However, within an 8 mm radius from the centre of the membrane, the strain forces remained largely homogenous. It has been previously shown that mechanical loading plays a major role in biochemical processes^2,6,17,29^. Additionally, mechanical strain of microtissues and cell cultures has been shown to result in the production of collagen with better mechanical properties than those of unstrained constructs^17^, improvement in the function of engineered cardiac tissue^29^, and promoted contractility and force maturation of cardiac constructs^6^.

This deflection was then coupled with the cyclic motion of the air-control valve, controlled by the DC motor and the Arduino (Fig. 1E). The repetitive motion of the valve controls the flow of air, which breaks the pressure differential within the device and creates a cyclic strain on the membranes; see video for a representative example of the membrane deflection here. The rate of deflection can be easily modified on the code of the Arduino, which allows for the simulation of different beating profiles such as tachycardia, normal heartbeat, bradycardia, and arrhythmia.Video S1. Representative video for BEaTS-alpha operating at 50 bpm. The video shows the vacuum-driven deflection of the membrane. No cell cells or cell culture media are present in the well. A yellow background is used to enhance the contrast of the video https://urldefense.proofpoint.com/v2/url?u=https-3A__www.dropbox.com_s_vqh4quhu9dlefv5_Video-2520Beating.mp4-3Fdl-3D0&d=DwMFaQ&c=vh6FgFnduejNhPPD0fl_yRaSfZy8CWbWnIf4XJhSqx8&r=W8RE-88OJ6HkLx5-vnSwGMbECZfnybKmwCXImBggWgpae5ASEwQeYcLKZnw2tz-i&m=tGgc2Rk-FybUSAPycWfc5XjlKdBZIdF1Hw76kkoSFaA&s=MKjZ5xrYMkdOM3XvqevS4oNd0xe5qe6Z1GBx_4NoXy4&e=.

#### Electrical stimulation

To electrically stimulate the cells, a C-PACE EP system was used. This device allows for the fine control of voltage, current, and frequency of the electrical pacing of cells seeded onto a substrate by means of two carbon electrodes that are placed inside the wells. In addition, it can be controlled externally using a transistor-transistor logic (TTL) signal and Bayonet Neil-Concelman (BNC) cables. The Arduino board used was programmed to create square TTL signals to control the electrical pacing of the cells. This allowed for the synchronization of both the electrical pacing and mechanical stimulation of the cells, creating a more physiologically relevant model. In addition, the compact design of the device allows for its use in both small and normal size incubators and the number of electrical and metal parts placed inside the incubator was minimized to avoid corrosion and overheating.

### Culture and immunostaining of human iPSC-derived cardiomyocytes (hiPSC-CMs)

The effects of the mechanical, electrical, and electromechanical stimulations on the differentiation and maturation of hiPSC-CMs were studied by culturing cells for 2, 4, or 7 days under each stimulation regime. All cells were seeded and pre-cultured for 24 hours before stimulation. hiPSC-CMs were then put under no stimulation (control), mechanical, electrical, or electromechanical stimulation for 2, 4, or 7 days at 37 °C, 5% CO_2_; cell media was changed daily. Cells in the mechanical group were stimulated in a normal heartbeat cycle (80 bpm), those in the electrical group were paced using the C-PACE EP system under 5 V/cm, 5 ms duration signals every 45 ms. This stimulation regime generated a current density of 13 mA cm^−2^ over the flexible membrane where the cells were seeded. The electromechanical groups were stimulated under a combination of both electrical and mechanical stimulation regimes using 80 bpm synchronic electric and mechanical deformation.

**Figure 2:**
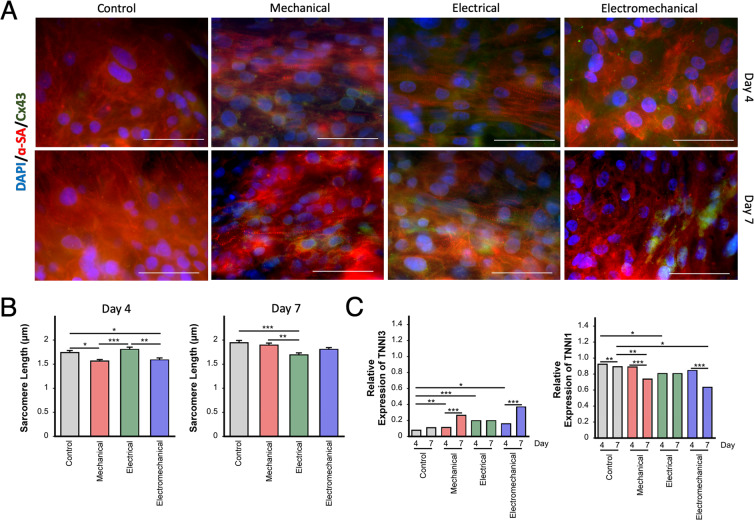
Effect of stimulation on hiPSC-derived cardiomyocyte cultures. (**A**) Representative immunofluorescent images of hiPSC-derived cardiomyocytes cultured on flexible silicone membranes under either no stimulation (control), mechanical stimulation, electrical stimulation, or mechanical and electrical stimulation at different time points (4 and 7 days). Cells were stained for α-sarcomeric actin (α-SA; red) and connexin-43 (Cx43; green). Nuclei were stained using DAPI (blue). Scale bars = 50 µm. (**B**) Effect of stimulus on sarcomere length measured at 4 and 7 days (n = 2). Values calculated from the measurement of 100 random cells over 6 different fields of view. (**C**) Relative gene expression for TNNI1 and TNNI3 measured for hiPSC cells cultured for 4 and 7 days without and with the different stimulation regimes (n = 3). *p < 0.05, **p < 0.01, and ***p < 0.001 calculated using One-way ANOVA with Tukey posthoc analysis.

Regardless of the stimulation regime, it was observed that all hiPSC-CMs expressed alpha-sarcomeric actin (α-SA), which is a key marker of cardiomyocytes (See Figs. 2 and S2). On day 2, a marked increase in the number of (Connexin-43) Cx43^+^/α-SA^+^ double-positive cells was observed for the electrical and electromechanically stimulated cells, which suggests that improved electrical connectivity improved maturation (Fig. S2B)^30^. Although the sarcomere structure was not well defined at this early timepoint, cells that were electromechanically stimulated presented a more anisotropic rod shape and an aspect ratio that approached 7:1, characteristic of mature human cardiomyocytes^30^. Further, analysis of the length to width ratio of the hiPSC-CMs after stimulation showed that electromechanically stimulated cells had an increased length-to-width aspect ratio with respect to all three other groups (2.3X fold of control and mechanically stimulated cells, and 3.6X fold of cells which were only electrically stimulated). Increasing the culture time to 4 and 7 days allowed for a clearer visualization of sarcomeric structures of the cells (Fig. 2A). Upon measuring the length of the sarcomeres, a significant decrease in length at days 4 and 7 for both the mechanical and electromechanical groups was observed. The electrically stimulated group showed an increase in sarcomere length on day 4, however, on day 7 the length decreased, see Fig. 2B. Our results are in agreement with those of Kroll *et al*.^1^, who reported that sarcomere length decreased under mechanical and electromechanical stimulation, resulting in shorter structures when compared to the non-stimulated control. They also found that electrical stimulation, using a C-PACE system, resulted in an increase of sarcomere length at day 3 but, as observed, this length decreased by day 7. In addition, qPCR analysis for the expression ratios of the cardiac (TNNI3) and skeletal (TNNI1) isoforms of troponin I^31^ showed an increase in expression of TNNI3 and a decrease in expression of TNNI1 for the mechanical and electromechanical groups at day 7 (Fig. 2C), a trend which further highlighted the maturation of the cells. This evidence suggests that the electromechanical stimulation regime provided by our device is capable of inducing maturation within hiPSC-CMs cultures. However, it is important to mention that these markers of maturation are only a fraction of those which have been previously explored in the examination of cardiomyocyte maturity and that other markers, such as Myosin Heavy Chain 7 (Myh7) and Sarcoplasmic/Endoplasmic Reticulum Ca^2+^-ATPase (SERCA) gene expression as well as contractile function have also shown promise. Given that there is no consensus in the field regarding what constitutes a mature cardiomyocyte^31^, we envision our low-cost, readily-available and open access device to be used as a tool to further study and develop these marker-maturity relationships. Furthermore, optimization of the mechanical and electrical stimulus timing and increases in the time of culture under stimulus could possibly enhance the observed maturation within our developed model.

## Conclusion

The current body of literature speaks to the clinical value of human-induced pluripotent stem cells as therapeutic agents for tissue regeneration and *in vitro* drug screening. As such, much research has been focused on the development of methods for maturing human cardiomyocytes from hiPSCs. However, despite recent advancements in cell culture techniques, which includes the incorporation of soluble factors, chemicals, and the use of 3-dimensional cultures; hiPSC-CMs typically only reach an immature state which hinders potential clinical translation and their use as physiologically relevant models for drug screening. Synchronic incorporation of non-linear mechanical deformation and electrical stimuli that recapitulates heart rhythm is thus critical to allow functional maturation of hiPSC cardiomyocytes.

BEaTS-α is a compact open-source system that incorporates elastic deformation capable of synchronic interfacing to a commercially available C-PACE-EP electrical stimulator to partially recapitulate the natural heart contraction and signal propagation. Our experimental indicates that hiPSC cells cultured under electromechanical stimulation showed a more mature cardiomyocyte phenotype than non stimulated cells. With its simple design and free access to code and CAD files for in-lab printing and assembly, we see this device as a low-cost and viable strategy for harmonizing the hiPSC-CMs differentiation protocols amongst labs. Through a simple modification of the code and hardware, it is also evident that BEaTS-α could also find applications in the *in vitro* study of other electro-responsive tissues such as muscles and nerves.

## Methods

### CAD modeling and 3D printing

All 3D printed components were designed using CAD software (SolidWorks, Dassault Systems) and 3D printed in an Ultimaker S5 (Ultimaker, Netherlands). The membrane holders (Fig. 1B) were printed in thermoplastic polyurethane (TPU 80 A) (Filaments Canada, Canada) while all other components were printed in Nylon 680 filament (Taulman, USA). Blueprints and files for printing this device are open-access and can be found via the following link. A detailed step by step guide for assembling and programming of the device is found in the Supporting information file of this article. Below, the key elements for assembling BEaTS-α are described.

### Finite element analysis (FEA)

The strain profile generated on the flexible membranes during the mechanical stretching was calculated through Finite Element Analysis performed on SolidWorks. To simulate the deflection of the membrane a model of the silicone was created and a material profile, using the material properties (AZO Materials), was applied. This model was then fixed at its ends simulating the fixed geometry when fully assembled in the membrane holder. A pressure of 55 kPa was applied at the bottom face of the membrane. The stress, strain, and displacement generated by the pressure differential across the membranes were calculated. A detailed description of the analysis and the stress and displacement profiles can be accessed in the Supplementary information (Section S1, ESI).

### Airflow control valve and plate holder assembly

Once the plate holder (Fig. S3), motor holder, and control valve parts were 3D printed, they were assembled prior to cell culture (Sections S2–S5, ESI). First, the 3D-printed valve, pin, seal, and nut that make up the control valve were assembled as shown in Figs. S4 and S5. To control the inflow of air, the valve pin was attached to the pin of the DC servo motor (Power: 4.8 V–6 V DC max, Torque: 8.5 kg-cm/10 kg-cm at 4.8 V and 6 V, respectively. Adafruit, USA). When the motor rotates the valve pin, air flows into the device, reducing the pressure applied on the flexible membranes and thus reduces the strain on the cells seeded on the membrane holders. Once having assembled the membrane holders (Fig. S6, ESI), and seeded the cells, the well plate was placed on the plate holder and sealed with and RTV108 medical-grade silicone molded seal (Fig. S6, ESI).

### Membrane holder assembly

The assembly of the membrane holders, plate holder, and additional sub-assemblies of the device was accomplished following the protocol provided in Section S6 (ESI). Briefly, the bottom of the wells of 6-well plates was removed with the aid of a punch and heating device and replaced with flexible, translucent silicone membranes. To assemble them, 25 mm squares were cut from a sheet of GraceBio labs silicone membrane (Grace Bio-labs, USA). The silicone squares were then placed on the bottom piece of the membrane holder (Fig. 1B) and fixed with RTV 108 medical-grade clear silicone (Mouser Electronics, USA). This subassembly was then glued to the top piece of the holder with RTV 108 silicone and allowed to cure overnight under sterile conditions (Fig. S7, ESI). After this curing process, each membrane was washed 4x with 70% ethanol and 5X with PBS.

### Arduino code

An Arduino UNO (Arduino, USA) was used to control the DC motor, to regulate the mechanical deflection of the membranes, and to control the C-PACE system. The code and circuitry used can be found in the Supplementary Information (Sections S7–S8 and Fig. S8 ESI). Briefly, to control the mechanical stimulation, the Arduino controls the rotation of the valve pin in the air-control system by the cyclic rotation of a DC servo motor. The code was set to 80 bpm; however, it can be easily modified to achieve different deflection rates as desired. To control the C-PACE EP system and thus the electrical pacing of the cells, the Arduino was programmed to produce TTL signals of 5 V which were sent to the C-PACE system through a BNC cable. The C-PACE EP system was programmed to the TTL lock modality as indicated by the manufactures manual (IonOptix, USA). The rate and duration of the TTL signal output of the Arduino can be modified on the code (ESI), however, the voltage, frequency, and duration of the pacing must be modified directly on the interface of the C-PACE EP system. In this experiment, TTL signals were sent every 45 ms with a duration of 5 ms (every time a TTL signal was sent as HIGH in the code), with a voltage of 5 V and a duration of 5 ms.

### Culture and differentiation of hiPSCs

All of the procedures were approved by the institutional animal care and ethical committee at the University of Ottawa. Investigations involving human tissues conform to the principles outlined in the Declaration of Helsinki, and informed consent was obtained from all subjects prior to their inclusion in the study. Human iPSCs were generated in Dr. Joseph Wu’s lab (Stanford University) with Sendai virus from peripheral blood mononuclear cells donated by a healthy volunteer (female, 41-year-old)^32^. Differentiation of hiPSCs into cardiomyocytes was performed using a 2-D monolayer protocol in a chemically defined medium^33^. iPSCs (passage 15–40) at 80–90% confluency were treated with 6 µM CHIR99021 (Selleck Chemicals) from day 0 to 2 in CDM3 medium (RPMI 1640 supplemented with 213 µg/mL L-ascorbic acid-2-phosphate and 500 µg/mL recombinant human albumin). Cells were maintained from day 2 to 4 in CDM3 medium containing 2 µM Wnt-C59 (Selleck Chemicals). At day 4, cells were cultured in control CDM3 medium (without other added factors) and spontaneous beating cells were observed under a microscope at day 7–10. Cells were glucose-starved from day 10 to 14 to purify cardiomyocytes. To minimize well-to-well variations during the differentiation, cells from different wells (at 4 weeks) were lifted with TrypLE (Life Technologies), pooled together, and then replated to the device for further culture at different testing conditions

Before segregating and reseeding the hiPSC cells onto the membranes, the modified wells were washed 4X with 70% ethanol and UV light treated for 45 minutes. They were then washed 5X with PBS. PBS was removed and 250 µL of 1X Attachment Factor (ThermoFisher, USA) was added to each membrane. All well plates were incubated at 37 °C, 5% CO_2_ for 2 h. To lift and reseed hiPSC onto the new membranes, cells were treated with Collagenase B in Ca^2+^-free Tyrode solution, pH = 6.9 (T6.9). First, 100 mg of Collagenase B was diluted in 1 mL of PBS, aliquoted to 50 µL in 0.6 mL Eppendorf tubes and kept at −80 °C. Two aliquoted solutions (100 µL) of the collagenase B/PBS solution was were taken out of the −80 °C freezer, thawed at room temperature, and diluted in 2 mL of T6.9. The hiPSC-CMs at day 30–40 of differentiation were washed gently with T6.9. Then, 500 µL of the diluted collagenase B solution was added to each well and the well plates were transferred to the cell incubator at 37 °C, 5% CO_2_ for 4 h. After 4 h, the collagenase B solution was quenched by adding 3 mL of cell culture media. Cells were collected in a 15 mL Falcon tube and spun at 200 G for 4 minutes. Approximately 7.5 × 10^5^ cells were seeded on each membrane and cultured for 24 hours before any stimulation was applied.

### Immunostaining and imaging

hiPSC-CMs were stained for DAPI, Connexin43 (Cx43), and alpha-sarcomeric actin (α-SA). Once the electrical, mechanical, and electromechanical stimulation were completed, the membrane’s holders were removed and the cells were fixed and stained for Cx43, α-SA, and DAPI. Briefly, cell media was removed from each well and then gently washed with PBS. Then, 500 µL of 4% PFA was added to each well and the plates were incubated at 4 °C for 15 minutes. PFA was then removed and 500 µL of blocking solution was added; wells were incubated at 4 °C for 30 minutes. The first antibody solution was prepared in diluting solution by adding mouse alpha-SA (1:400, Sigma) and rabbit connexin43 (1:200, Sigma). To each well with cells, 1 mL of this solution was added; well plates were incubated at 4 °C overnight. The next day, working in darkness and after removing the first antibody solution, 500 µL of the second antibody solution containing anti-rabbit Alexa Fluor 488 (1:600, ThermoFisher), anti-mouse Alexa Fluor 546 (1:600, ThermoFisher), and DAPI (1:100) were added to each well. Cells were incubated at room temperature for one hour.

After fixation, the centre of the membranes (8 mm) were perforated and the membrane mounted on microscope slides and imaged with a fluorescence microscope (Zeiss Axiovert 200 M fluorescence microscope equipped with an AxioCam MR camera. DAPI blue filter Ex: 352–402 nm / Em: 417–477 nm. GFP green filter Ex: 457–487 nm / Em: 502–538 nm. Texas red filter Ex: 542–582 nm / Em: 604–644 nm). Images were taken from 3–4 random regions. The images were analyzed using ImageJ software (National Institutes of Health, USA) (see Fig. 2).

### Sarcomere length

Sarcomere length was determined by measuring the banding pattern of the α-SA signal, in the red channel of the immunostained images, using the ROI line and plot profile tools built into ImageJ software (National Institutes of Health, USA). Values were calculated from the measurement of 100 random cells over 6 different fields of view.

### qPCR of TNNI1 and TNNI3

hiPSC-CMs were cultured for 4 and 7 days under the various conditions at which point total RNA was isolated using the RNeasy kit (Qaigen). First-strand cDNA was synthesized with Smartscribe Reverse Transcriptase (Takara Bio USA) and random hexamer primers (Fisher Scientific). Target gene mRNA levels were assessed by quantitative RT-PCR with LightCycler 480 SYBR Green I Mastermix (Roche) and a LightCycler 480 Real-Time PCR System (Roche). Relative changes in mRNA expression were determined by the ∆∆Ct method, expressed as levels relative to 18 S.

### Statistical analysis

Statistical analysis was carried out using One Way ANOVA with Tukey posthoc analysis in Kaleida 4.5.

## Supplementary information

Supplementary Information.

## Data Availability

All data generated or analyzed in this study are included in the manuscript and the Supplementary materials. The data used to produce the figures shown in this article, including the LTS file for the device, are available at https://figshare.com/s/504b0f7ccbdc7b893e3a. The experimental data that support the findings of this study are available from the authors.
