# Supplementary material for: BEaTS-α an open access 3D printed device for in vitro electromechanical stimulation of human induced pluripotent stem cells
Source: Sci Rep. 2020 Jul 9;10:11274. doi: 10.1038/s41598-020-67169-1 (PMC7347879; doi:10.1038/s41598-020-67169-1)
Supplement: Supplementary file 1 — Supplementary Information. [file 41598_2020_67169_MOESM1_ESM.pdf]

Electronic Supplementary Information (ESI) for:

BEaTS-α an open access 3D printed device for *in vitro* electromechanical stimulation of human induced pluripotent stem cells

David Cortes[1,2†], Christopher D. McTiernan[2†], Marc Ruel[2], Walfre Franco[3], Cencen Chu[4,5], Wenbin Liang[4,5], Erik J. Suuronen[2,5], and Emilio I. Alarcon[2,6*]

[1]Biomedical Mechanical Engineering, University of Ottawa, Ottawa, ON, K1N6N5, Canada
[2]Division of Cardiac Surgery, University of Ottawa Heart Institute, Ottawa, ON, K1Y4W7, Canada
[3]Wellman Center for Photomedicine, Department of Dermatology, Massachusetts General Hospital, Harvard Medical School, Boston, MA, 02114, USA
[4]Cardiac Electrophysiology Laboratory, University of Ottawa Heart Institute, Ottawa, ON, K1Y4W7, Canada [5]Department of Cellular and Molecular Medicine, University of Ottawa, Ottawa, ON, K1H8M5, Canada
[6]Biochemistry, Microbiology and Immunology, University of Ottawa, Ottawa, ON, K1H8M5, Canada
*email: ealarcon@ottawaheart.ca
†These authors contributed equally to this work

Table of Contents

S1. Finite Element Analysis of membranes 3

S2. Assembly manual – general instructions 5

S3. Plate holder assembly 6

S4. Airflow control valve assembly 7

S5. Final assembly of electromechanical stimulation device and cleaning procedure 9

S6. Membrane holder assembly 10

S7. Arduino code 12

S8. Circuits 12

S8.1 C-PACE EP system 13

S1. Finite Element Analysis of membranes

A model of the silicone membranes was generated in CAD (SolidWorks) and the stress, strain, and displacement profiles of the membranes were calculated. A profile for the material of the membranes was created using the material specifications. The silicone was modeled as linear elastic isotropic, with the following properties:

i. Elastic Modulus: 50 MPa
ii. Poisson's ratio: 0.49
iii. Shear Modulus: 20 MPa
iv. Mass density: 2300 kg/m^3
v. Tensile strength: 5.5 MPa
vi. Compressive strength: 70 MPa

Once the material was applied, the model was fixed at the borders simulating the fixtures when the membrane is assembled in the membrane holders, and a pressure of 55 KPa was applied to the bottom face of the sheet. The average stress (Von Mises), displacement (URES), and strain (Figure S1) were calculated to be 1.249 kPa, 0.545 mm, and 2.34%, respectively.

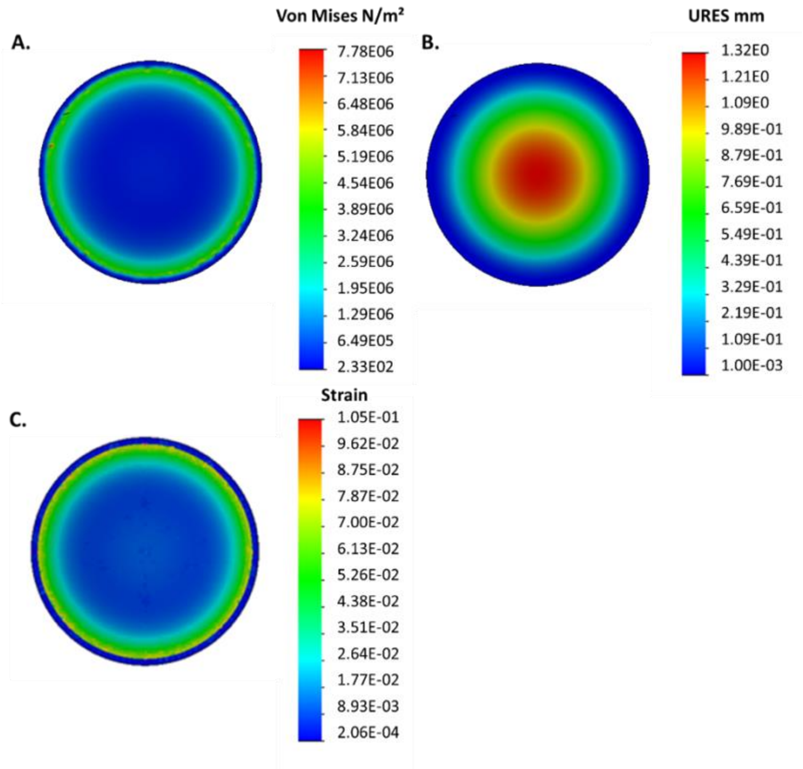

Figure S1. Finite Element Analysis (FEA) of stress, displacement, and strain profile of silicone membrane under mechanical loading generated by the pressure differential (55kPa) during the cyclic mechanical stimulation. Average stress, displacement, and strain were calculated as 1.249 kPa, 0.545 mm, and 2.34%, respectively.

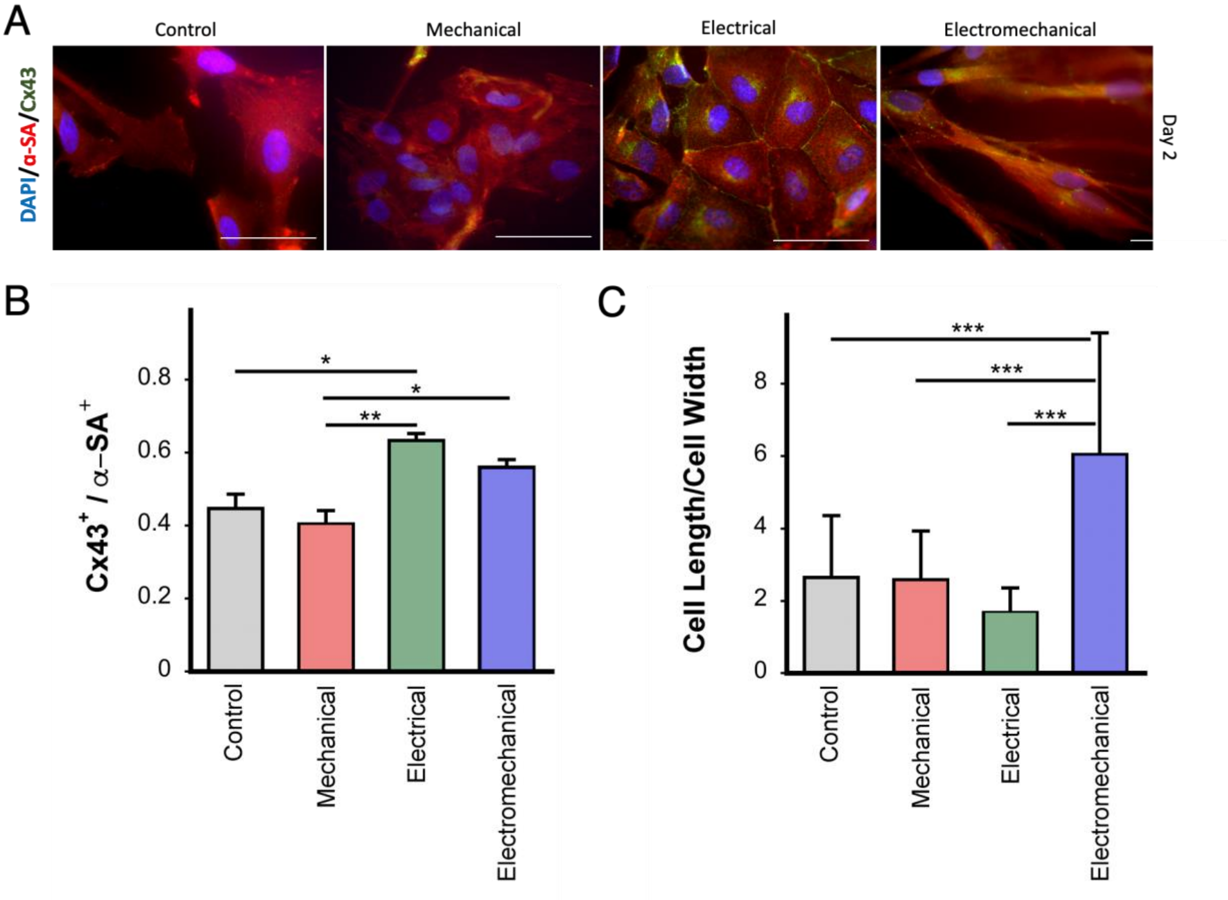

Figure S2. Effect of stimulation on hiPSC-derived cardiomyocyte cultures. (A) Representative immunofluorescent images of hiPSC-derived cardiomyocytes cultured on flexible silicone membranes under either no stimulation (control), mechanical stimulation, electrical stimulation, or mechanical and electrical stimulation for 2 days. Cells were stained for α-sarcomeric actin (α-SA; red) and connexin-43 (Cx43; green). Nuclei were stained using DAPI (blue). Scale bars = 50 µm. (B) Effect of stimulus on the expression of Cx43 within hiPSC-derived cardiomyocyte cultures. Values are expressed as the ratio between Cx43/α-SA double-positive cells relative to α-SA single positive cells. (C) Effect of stimulus on the cell length to cell width ratio within hiPSC-derived cardiomyocyte cultures. Error bars correspond to the standard error of the mean (n=3). *$p<0.05$, **$p < 0.01$, and ***$p<0.001$ calculated using One-way ANOVA with Turkey posthoc analysis.

S2. Assembly manual – general instructions

i. All components must be 3D printed with the appropriate materials as listed in Table S1 prior to the assembly of the device. All files are accessible here as .stl files. It should be noted that all components printed with Nylon 680 materials were set up with a custom material profile created in Cura.

ii. An Ultimaker S5 printer was used to 3D print all components. The use of an Ultimaker is not mandatory; however, tolerances might be subject to change depending on the resolution of the printer and the quality of the materials used.

Table S1. List of .stl files accessible for 3D printing as Cura projects

**Part name**	**Quantity**	**Material**	**Printing profiles**
Plate holder	1	Nylon 680	A
Motor base	1	Nylon 680	A
Motor pin	2	Nylon 680	A
Valve retainer	1	Nylon 680	A
Valve	1	Nylon 680	A
Drafted valve pin	1	Nylon 680	A
Nut	1	Nylon680	A
Seal	1	Nylon 680	A
Seal mold-extruded	1	PLA	B
Seal mold-hole	1	PLA	B
Membrane holder bottom	1*	TPE 80A	C
Membrane holder top	1*	TPE 80A	C

* The number of membrane holders to be printed will vary depending on the experiment set up; a membrane holder assembly consists of *Membrane holder bottom x1, Membrane holder top x2,* and a translucent silicone sheet (see S5 for details).

Printing profiles:

A. Nylon 680:

- Nylon 680 Cura material profile:
  - Display name: Nylon 680
  - Brand: Custom
  - Material type: Nylon
  - Density: 1.14 g/cm^3
  - Diameter: 2.85 mm
  - Adhesion information: Print on bare glass. Use tape, glue, or spray glue to enhance adhesion.
- Printing specifications:
  - Layer height: 0.2mm
  - Wall Thickness: 2.0
  - Top/Bottom thickness: 2.5
  - Infill density: 35%
  - Infill pattern: triangles
  - Printing temperature: 255°C
  - Build plate temperature: 60°C

- Print speed: 70 mm/s
- Travel jerk: 50 mm/s
- Support: Touching build plate (overhang angle 60)

B. PLA

- PLA material profile: Generic PLA profile (Cura)
- Printing specifications:
  - Layer height: 0.15mm
  - Wall thickness: 1.2mm
  - Top/Bottom thickness: 1.2mm
  - Infill density: 20%
  - Infill pattern: Triangles
  - Printing temperature: 200°C
  - Build plate temperature: 60°C
  - Enable retraction
  - Print speed: 70 mm/s
  - Travel jerk: 50 mm/s
  - Support: Everywhere (overhang angle 60)

C. TPE 80A

- Material profile: Generic TPU 95A
- Printing specifications:
  - Layer height: 0.15mm
  - Wall thickness: 0.8mm
  - Top/Bottom thickness: 0.8mm
  - Infill density: 40%
  - Infill pattern: Concentric
  - Printing temperature: 235°C
  - Build plate temperature: 80°C
  - Do not enable retraction
  - Print speed: 15 mm/s
  - Travel jerk: 30 mm/s
  - Support: Everywhere (overhang angle 50)

iii. Before assembling and *in vitro* experiments, rinse all materials 4X with 70% ethanol and sterilize for a minimum of 45 minutes under UV light. Cleaning procedures for membrane holders are explained in depth in section *S5. Membrane holder assembly*.

S3. Plate holder assembly

i. Materials

a. Plate holder
b. Motor base
c. Valve retainer
d. Valve
e. Drafted valve pin
f. Nut
g. Seal
h. Mold for RTV seal
i. RTV 108 clear silicone

j. DC servo motor (Power: 4.8V – 6V DC max, Torque: 8.5 kg-cm/10 kg-cm at 4.8V and 6V, respectively. Adafruit, USA)
k. 70% ethanol
l. Laboratory tubing (Fisherbrand, OD= ½ in, ID= 3/8 in)

ii. Assembly protocol:

a. A representative scheme of the plate holder assembled is depicted in Figure S3.
b. Once having 3D printed all necessary components, slide the motor base (E) into the slots of the plate holder (A) until the holes are aligned.
c. Insert the two pins (B) into the holes to secure the motor base.
d. Place the DC motor (D) at the top of the motor base and secure it using the screw set (C) provided by the seller of the DC motor by screwing the bolts through the holes od both the DC motor and the motor base.

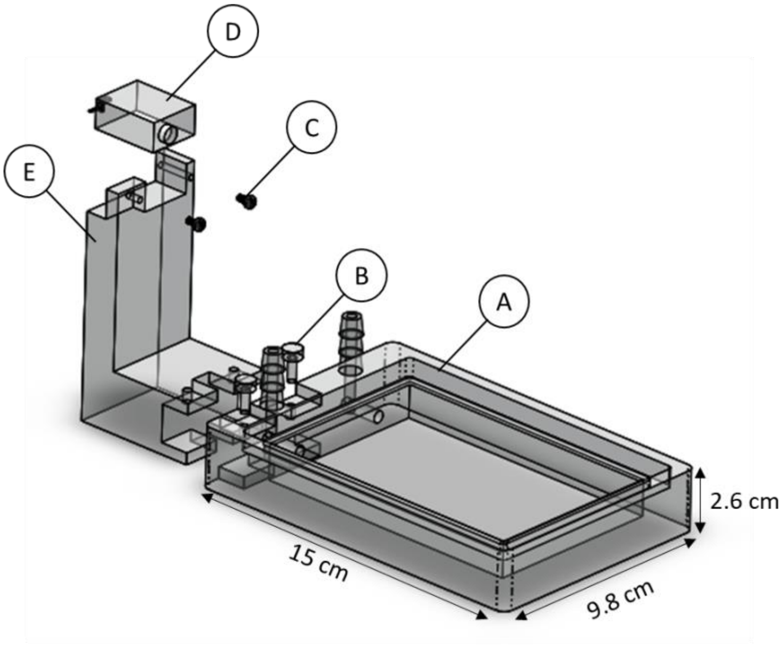

Figure S3. Scheme of plate and motor holder assembly. Parts A) plate holder, B) motor pin, and E) were 3D printed with Nylon 680 filament in and Ultimaker S5. The DC motor (D) and screw set (C) were purchased with the Arduino UNO motherboard (Arduino, USA).

S4. Airflow control valve assembly

i. Materials:

a. Valve retainer
b. Valve
c. Drafted valve pin
d. Nut
e. Seal
f. Laboratory tubing

ii. Assembly protocol of airflow control valve:

a. All the parts of the airflow control valve were 3D printed in an Ultimaker S5 with a 0.4 mm extrusion nozzle and using Nylon 680 filament with the specifications provided for

printing profile A. The tolerances and quality of the prints are subject to change if different materials or 3D printers are used.

b. After 3D printing all components required for the valve assembly, the airflow control valve must be assembled as shown in Figure S4.
c. First, insert the valve pin (F) into (G). Make sure that when (F) is rotated, the hole aligns with that of Part (G). This hole will allow the air inflow during the mechanical stimulation.
d. Place the 3D printed seal (H) and screw (I) gently to secure the valve pin (F) yet allowing it to rotate inside of (G).

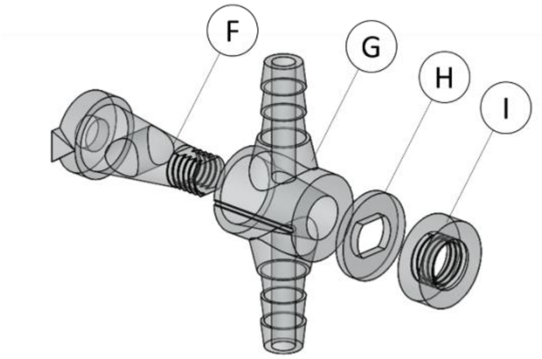

Figure S4. Airflow control valve. Part (F) is controlled by the DC servo motor (Figure S1, D). When (F) is rotated approximately 35° (Arduino code), the hole aligns with that of part (G) allowing the flow of air into the plate assembly. The cyclic flow of air is used to create a cyclic deflection of the membranes which results in the mechanical stimulation of the cells. Parts (H) and (I) are used to secure part (F) and create a thigh seal.

iii. Assembling *airflow control valve* and *plate motor holder assembly* together:

a. To assemble the *airflow control valve*, place a 2cm long piece of laboratory tubing (J) around the inflow valve of the plate holder as shown in Figure S5a.
b. Before inserting the *airflow control valve* in the tubing, slide the valve retainer (K) around the tubing and clamp the ends of it around the motor base as shown in Figure S5b.
c. Then, insert one end of the control valve into the tubing as shown in Figure S5a.

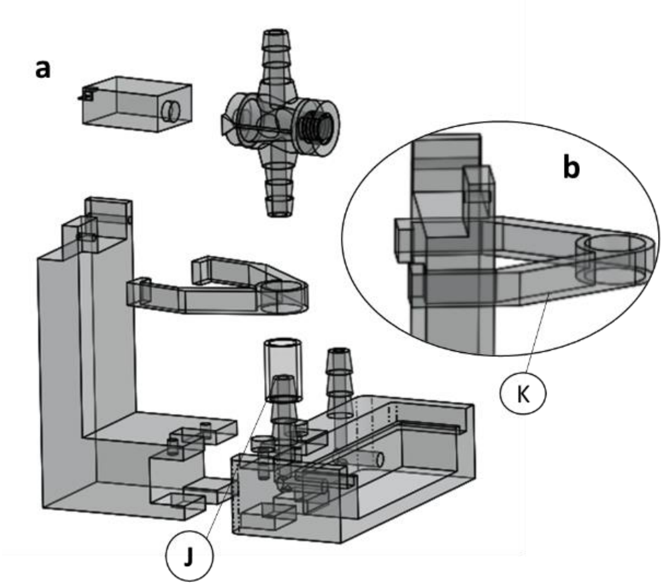

Figure S5. Assembly of the airflow control valve, plate holder, and valve retainer. An exploded-view (4a) of the components shows the tubing (J), valve retainer (K) and valve assembly in the order they must be assembled. Before inserting the airflow control valve assembly, the valve retainer must be placed around (J) and clamped on the motor base as shown in detailed view b.

S5. Final assembly of electromechanical stimulation device and cleaning procedure

i. Materials:
   a. Subassemblies:
      i. Plate holder assembly
      ii. Airflow control valve assembly
      iii. Membrane holder assembly
   b. Mold for RTV seal
   c. RTV 108 medical-grade silicone
   d. 70% ethanol
   e. Cell hood with UV light
   f. 1x Phosphate buffer solution (PBS)

ii. Cleaning protocol:
   a. Once having assembled the plate holder and the airflow control valve as described above, clean the device with 70% ethanol and sterilize it under UV light for a minimum of 60 minutes.
   b. To prepare the 6-well plates for cell culture, assemble the desired number of membrane holders as described in section S5.
   c. Rinse each modified well 4 times with 70% ethanol.
   d. Rinse the wells with 1x PBS 5 times.
   e. Sterilize the well plates under UV light for a minimum of 120 minutes.

iii. Final assembly of electromechanical stimulation device:
   a. Before placing the 6-well plate, a silicone seal must be cast using the *Mold for RTV seal* PLA mold and RTV 108 medical-grade silicone.

b. To create the seal, place together both sides of the seal mold by inserting the extruded pin onto the hole on the mirror part of the mold (Figure S7a).
c. Once assembled, fill the mold with enough RTV 108 silicone avoiding creating any bubbles.
d. Allow the silicone cure for a minimum of 24 hours. Once the silicone is completely cured, carefully disassemble the molds. With the help of a spatula, remove the silicone seal from the molds.
e. Once all the parts have been 3D printed and assembled into the subassemblies described above, place the well plate in the plate holder.
f. Locate the silicone seal around the motherboard plate to create an air-thigh seal.
g. At this point, the device can be placed inside the cell incubator to begin the stimulation of the cells.

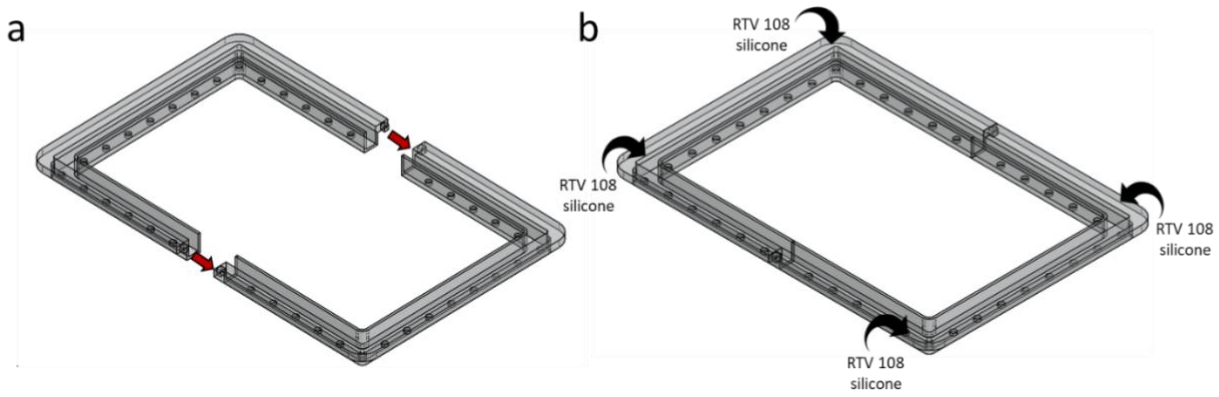

Figure S6. 3D printed mold used for casting the silicone seal used in the final assembly of the electromechanical stimulation device.

S6. Membrane holder assembly

i. Materials:
   a. Membrane holder bottom
   b. Membrane holder top
   c. Translucent silicone membranes - 0.5mm thickness (GraceBiolabs)
   d. RTV 108 medical-grade silicone
   e. 20mm histology metal punch
   f. 3mm histology punch
   g. Torch (handheld/baker)
ii. Membrane holder assembly protocol:
   a. The number of membrane holders to be assembled will depend on the experiment protocol and the type of stimulation the cultured cells will be put under. Each membrane holder is to be placed in the bottom of a well on a 6-well plate (Figure S7).
   b. Prior to starting the assembly of each holder, the *Membrane holder bottom* and *Membrane holder top* files must be 3D printed with TPU 80A filament.

c. Using the torch and the 20 mm metal histology punch, remove the bottom of the wells that are to be used for mechanical/electromechanical stimulation as shown in Figure S7b(2).
d. Once having 3D printed both top and bottom parts of the holders and having removed the bottom of the wells, cut a square of 25.4 mm from the translucent silicone sheet (Figure S7b(1)).
e. Cover the bottom membrane holder (N) with RTV108 medical-grade silicone such that the silicone reaches the edge of the holders.
f. Carefully place the silicone sheet on top of the silicone ensuring all sides are aligned (Figure S7b(2)).
g. With the 3mm histology punch, cut 3mm circles at the edges of the silicone sheet. It is recommended to allow the silicone to cure for a minimum of 20 minutes before continuing with the next step.
h. Insert the top part of the membrane holders into the wells of the 6-well plates (Figure S7b(3)).
i. Carefully add another layer of RTV 208 silicone on top of the silicone membrane, ensuring no RTV spreads towards the middle of the silicone square. It is recommended to cut tape in circles of 17mm of diameter and place them in the middle of the sheet as depicted in Figure S7(b) to prevent the silicone from spreading into the middle where cells will be seeded.
j. Place and lightly press the bottom subassembly (parts M and N) on the top part (L) making sure all sides align (Figure S7b(4)).
k. Carefully add another layer of silicone around the borders of the membrane holder assembly.
l. Allow the silicone to cure for a minimum of 2 hours, the silicone should be completely cured after 24h.
m. Once the silicone has been cured, carefully remove the circle tape from the silicone sheets.
n. Repeat this process for every membrane holder that is to be assembled.

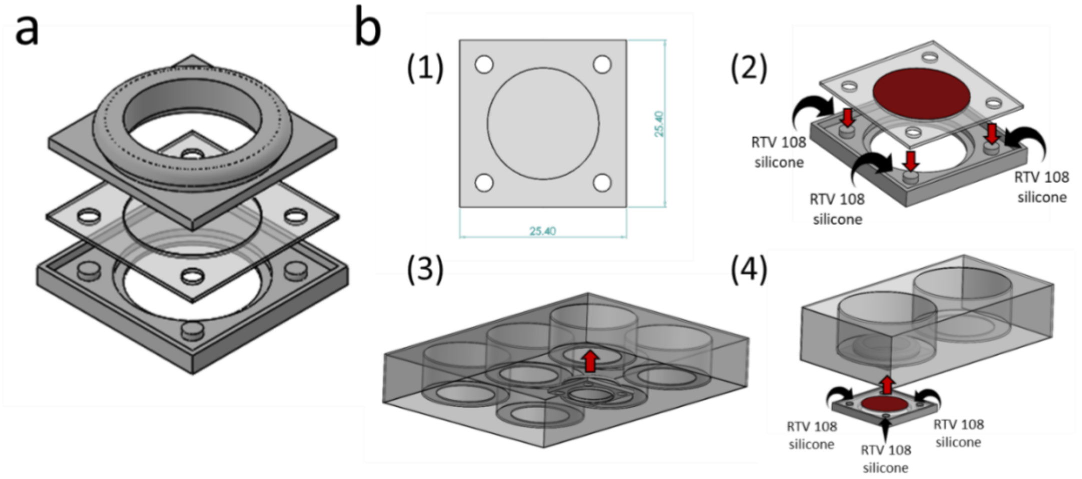

Figure S7. Assembly of membrane holders for the electromechanical stimulation of cells. A) Membrane holder exploded view depicting the top and bottom membrane holder parts, and the silicone membrane where cells were seeded. B) Steps for the assembly of the membrane holders.

S7. Arduino code

i. The Arduino file containing the code to control the DC servo motor and the C-PACE EP system can be found here.
ii. The code can be modified to vary the rhythm profile of both mechanical and electrical stimulation. This allows the user to create stimulation profiles simulation constant cyclic stimulation, arrhythmia-like cycles, tachycardia and bradycardia, mechanical-only, or electrical-only stimulation profiles.

S8. Circuits

i. Materials:
    a. Arduino UNO
    b. Jumper cables
    c. Male BNC Arduino connector
    d. Mini breadboard
    e. DC servo motor
    f. Male-to-male BNC cable
    g. C-PACE EP system
    h. Arduino code (S7)
    i. Electromechanical stimulation device assembled (S5)
ii. Before beginning the wiring of the system, upload the *Arduino code* provided in section S6 into your Arduino UNO.
iii. Figure S8 shows a schematic of the circuit that controls the C-PACE EP system and the DC motor.
iv. First, connect the 5V outlet of the Arduino to the mini breadboard.
v. Connect the Ground outlet to a parallel line on the same breadboard.
vi. Using another jumper wire, connect the 5V cable of the DC servo motor (red) to the 5V connection from the Arduino to the breadboard (iv).
vii. Connect the ground cable of the DC servo motor (black) the Ground connection from the Arduino to the breadboard (v).
viii. Connect the input cable of the DC servo motor (yellow) to pin 9 on the Arduino UNO.
ix. Connect a jumper from the ground line on the breadboard (v) to one of the pins on the BNC male Arduino connector.
x. Connect the other pin of the BNC male Arduino connector to pin 8 on the Arduino UNO.
xi. Using the male-to-male BNC cable, connect the BNC male Arduino connector to one of the male BNC inlets on the C-PACE EP System. Refer to section S8.1 for instructions on how to set-up the C-PACE EP system for TTL lock mode.
xii. Using the cable provided by the manufacturer, power the Arduino UNO board connecting it to a computer/power outlet.
xiii. Insert the pin of the DC servo motor on the *valve pin*. Make sure that the “arrow” of the *valve pin* lines up with the line on the *valve*, and that when the pin is rotated, the hole of the pin can be seen from the top outlet of the valve.

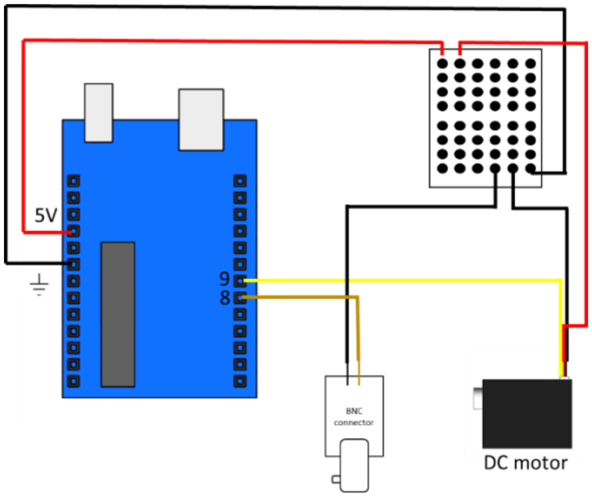

Figure S8. Circuit connection of the electromechanical stimulation device. The Arduino UNO board controls the cyclic rotation of the DC motor and the output of a TTL square signal to control the C PACE EP system.

S8.1 C-PACE EP system

i. TTL Lock mode set-up:
   a. For more detailed information on how to set up the C-PACE EP system please refer to the manufactures manual.
   b. Briefly, after connecting the BNC male Arduino connector to the C-PACE EP system, select the channel that will be used on the C-PACE EP interface.
   c. Using the knob, select “TTL Lock” on the first line.
   d. Choose the duration and voltage the system will apply every time there is an external TTL HIGH signal (from the Arduino).
   e. Choose “Enable” on the last line of the interface to turn on the channel.

ii. Please follow the instructions for cleaning the C PACE electrodes before and after usage provided by the manufacturer.
